# Primary replication and invasion of the bovine gammaherpesvirus BoHV-4 in the genital mucosae

**DOI:** 10.1186/s13567-017-0489-3

**Published:** 2017-11-28

**Authors:** Bo Yang, Yewei Li, Osvaldo Bogado Pascottini, Jiexiong Xie, Ruifang Wei, Geert Opsomer, Hans Nauwynck

**Affiliations:** 1 Department of Virology, Parasitology and Immunology, Faculty of Veterinary Medicine, Ghent University, Salisburylaan 133, B-9820 Merelbeke, Belgium; 2 Department of Reproduction, Obstetrics and Herd Health, Faculty of Veterinary Medicine, Ghent University, Salisburylaan 133, B-9820 Merelbeke, Belgium

## Abstract

Bovine herpesvirus 4 (BoHV-4) is a gammaherpesvirus that is widespread in cattle. Ex vivo models with bovine genital tract mucosa explants were set up to study molecular/cellular BoHV-4-host interactions. Bovine posterior vagina, cervix and uterus body were collected from cows at two stages of the reproductive cycle for making mucosa explants. The BoHV-4 replication kinetics and characteristics within the three different mucosae of animals in the follicular and luteal phase were assessed by virus titration. The number of plaques, plaque latitude and number of infected cells were determined by immunofluorescence. BoHV-4 replicated in a productive way in all genital mucosal tissues. It infected single individual cells in both epithelium and lamina propria of the genital mucosae at 24 hours post-inoculation (hpi). Later, small BoHV-4 epithelial plaques were formed that did not spread through the basement membrane. 50% of the number of BoHV-4 infected cells were identified as cytokeratin^+^ and CD172a^+^ cells in the three parts of the genital tract at 24 hpi. Upon a direct injection of genital explants with BoHV-4, fibrocytes became infected, indicating that the unidentified 50% of the infected cells are most probably fibrocytes. In this study, in vivo-related in vitro genital tract models were successfully established and the early stage of the pathogenesis of a genital infection was clarified: BoHV-4 starts with a productive infection of epithelial cells in the reproductive tract, forming small foci followed by a non-productive infection of surveilling monocytic cells which help BoHV-4 to invade into deeper tissues.

**Electronic supplementary material:**

The online version of this article (10.1186/s13567-017-0489-3) contains supplementary material, which is available to authorized users.

## Introduction

Bovine herpesvirus 4 (BoHV-4) is a gammaherpesvirus belonging to the genus *rhadinovirus*. It has been related with several diseases in cattle. BoHV-4 was first isolated from bovine with respiratory problems and keratoconjunctivitis in 1963 in Hungary [1]. Since then, the virus has been isolated from cattle with respiratory disease, conjunctivitis, metritis, ulcerative mammillitis, dermatitis and abortion in Europe, North America, Africa and Asia [2–6]. However, it has also been isolated from healthy cattle [7]. Up till now, little is known on how BoHV-4 may cause disease in bovine.

Uterine infections can cause significant economic losses in cattle industry. Although most causative agents have been identified as bacteria, BoHV-4 has been considered as a possible (co-)factor in post-partum metritis [8]. BoHV-4 is widespread in bovine and remains latent and asymptomatic in the vast majority of infected individuals. BoHV-4 persists mainly in peripheral blood leukocytes, the nervous system and lymphoid organs [9, 10]. Until now, a lot of work has been done to acquire more information on the viral molecular biology of BoHV-4. There are some reports on viral entry [11–13] and immune evasion [14] of BoHV-4. In addition, the construction of a BoHV-4 bacterial artificial chromosome (BAC) has provided an efficient tool for the study of the viral molecular biology of BoHV-4 [15–19]. Jacca et al. forwarded a hypothesis about the pathogenesis of BoHV-4: bovine macrophages are latently infected with BoHV-4 and upon reactivation, bystander endometrial stromal cells become infected [21]. This replication in the endometrium may cause inflammation. Once there, BoHV-4 might initiate replication within macrophages and spread to endometrial stromal cells, which are highly susceptible for BoHV-4 [20, 21]. However, data concerning the early pathogenesis of BoHV-4 at the genital tract are scarce. One of the biggest obstacles in getting better insights is the lack of in vivo-related in vitro models. Therefore, we developed in vitro models to examine the early BoHV-4 replication in the genital tract.

## Materials and methods

### Virus strain

The BoHV-4 strain V.test was used in this study, which belongs to the European clade of BoHV-4 strains. It was originally isolated from an infertile bull’s testicle [17]. The strain V.test had previously received an unknown number of passages. The virus was passaged two times in Madin-Darby Bovine Kidney (MDBK) cells in our laboratory.

### Animals

Healthy genital tissues (posterior vagina, cervix and uterus body) from cows of 2–4 years old were collected in a local slaughterhouse. In addition, blood was collected to determine BoHV-4 specific neutralizing antibodies with a complement-dependent seroneutralization (SN)-test and blood progesterone (P4). The stage of the reproductive cycle was determined by a morphological analysis of the ovaries and the blood progesterone (P4) level.

### Preparation of air–liquid interface tissue culture

From three cows in the luteal phase and three cows in the follicular phase, posterior vagina, cervix and uterus body were collected at the local abattoir immediately after slaughter. The cultivation protocol of bovine genital mucosa was performed as described before [22–24]. In brief, genital tissues of cows were immediately placed in phosphate buffered saline (PBS), supplemented with 1000 U/mL penicillin (Continental Pharma, Puurs, Belgium), 1 mg/mL streptomycin (Certa, Braine l’Alleud, Belgium), 1 μg/mL gentamycin (Invitrogen, Paisley, UK) and 5 μg/mL fungizone (Bristol-Myers Squibb, New York, USA) on ice for transportation to the laboratory. The mucosae from vagina, cervix and uterus body were stripped from the underlying layers. Afterwards, tissues were cut into small equal square pieces (on average 25 mm^2^). Finally, genital mucosa was placed on sterilized gauzes in 6-well plates for culture. The explants were cultured in serum-free medium [50% DMEM (Invitrogen)/50% Ham’s F-12 Gluta-MAX (Invitrogen)], supplemented with 100 U/mL penicillin (Continental Pharma), 0.1 mg/mL streptomycin (Certa) and 1 μg/mL gentamycin (Invitrogen) for up to 96 h (37 °C and 5% CO_2_).

### Evaluation of tissue viability

All tissues were monitored by measuring the occurrence of apoptosis to determine the viability during in vitro culture. The In Situ Cell Death Detection Kit (Roche Diagnostics, Switzerland) based on terminal deoxynucleotidyl transferase dUTP nick end-labeling (TUNEL) was used to evaluate the DNA fragmentation. The test was performed according to the manufacturer’s guidelines. TUNEL-positive cells were counted from five randomly chosen fields of 100 cells in the epithelium as well as in the lamina propria at 0, 24, 48, 72 and 96 h of culture. All stainings were analyzed with a TCS SPE confocal system (Leica Microsystems GmbH, Wetzlar, Germany).

### Virus inoculation

After 24 h cultivation of explants, the inoculation was performed. Genital explants were taken from their gauze and placed in a 24-well plate. After 2 washings with PBS, explants were either submerged in 0.5 mL of BoHV-4 containing medium (10^7^ TCID_50_/mL) and incubated for 1 h (37 °C, 5% CO_2_) or mock inoculated. Before explants were placed back on the gauze, they were washed three times with PBS. The inoculated tissues were collected at 0, 24, 48 and 72 hpi. In addition, explant cultivation medium was also collected for virus titration. All gathered explants were embedded in cryoprotection medium [Methocel^®^, Fluka (Sigma)] and then frozen at −70 °C.

### Replication kinetics of BoHV-4 in the mucosa of different parts of the genital tract

#### Virus titration

Explant cultivation medium was collected at 0, 24, 48 h and 72 hpi from BoHV-4 inoculated and mock inoculated explants for virus titration. Briefly, MDBK cells were inoculated for 1 h (37 °C, 5% CO_2_) with serial tenfold dilutions (10^0^ to 10^−7^ in quadruplicate) of BoHV-4 and mock inoculated explant medium. Afterwards, MDBK cells were observed daily for cytopathic effect (CPE) for 7–9 days.

#### Evaluation of primary viral replication in the genital mucosae

At 0, 24, 48 h and 72 hpi, mucosa explants were collected. To evaluate the lateral spread of BoHV-4 in the epithelial cell layer and its penetration in the connective tissue underneath the basement membrane, a double staining was performed. Cryosections (20 μm) of the different explants were made, paraformaldehyde fixed for 15 min at 4 °C and then permeabilized with PBS containing 0.1% Triton X-100 (PBST) for 10 min. After three washings, a primary mouse monoclonal IgG2a antibody (Mab35) against the glycoprotein complex gp6/gp10/gp17 of BoHV-4 (1:1000 in PBS) was used [25–27]. Next, a secondary goat anti-mouse IgG2a Alexa fluor^®^ 488 (Invitrogen) (4 μg/mL) was used. Analyses of IF stainings were performed by TCS SPE confocal system (Leica Microsystems GmbH, Wetzlar, Germany). Thereafter, the plaque latitude was evaluated using the line-tool function of the software program ImageJ.

#### Quantification and characterization of BoHV-4 infected cells

In order to identify the single BoHV-4 infected cells early after inoculation, double immunofluorescence stainings were performed using different cell surface markers. At least 20 cryosections (20 μm) were made for each marker of both BoHV-4 and mock inoculated genital mucosae. Cryosections were fixed in methanol (−20 °C, 100%) for 20 min and washed in PBS. Afterwards, they were incubated with a primary mouse monoclonal IgG2a antibody (Mab35) against BoHV-4 for 1 h at 37 °C, followed by an incubation with a secondary goat anti-mouse IgG2a Alexa fluor^®^ 594 (Invitrogen) (4 μg/mL). Afterwards, a primary monoclonal mouse IgG1 antibody DH59B (VMRD Inc., Pullman) or monoclonal mouse IgG1 anti-human cytokeratin (Dako, Glostrup, Denmark) (1.72 μg/mL) with a secondary goat anti-mouse IgG1 FITC^®^ (Abcam, Cambridge, UK) (1 μg/mL) were used to stain monocytes/macrophages/dendritic or epithelial cells, respectively. A polyclonal sheep anti-bovine IgM labeled with FITC^®^ (AbD Serotec) (6 μg/mL) was used to stain B lymphocytes. A primary monoclonal rat IgG1 anti-human CD3 (AbD Serotec, Biorad-laboratories, Kidlington, UK) (10 μg/mL) and secondary goat anti-rat Alexa fluor^®^ 488 (Invitrogen) (2 μg/mL) were used to stain T lymphocytes. Moreover, a primary monoclonal mouse IgG1 anti-vimentin (AbD Serotec) (1:100 in PBS) with a secondary goat anti-mouse IgG1 FITC^®^ (Abcam, Cambridge, UK) (1 μg/mL) were used to detect cells of mesenchymal origin. All incubation steps were performed at 37 °C for 1 h followed by three washings. Finally, Hoechst 33342 (Invitrogen) (10 μg/mL) was used in the last step to stain cell nuclei at 37 °C for 10 min. After washing with PBS, the cryosections were mounted with glycerin-DABCO and then analyzed by TCS SPE confocal system (Leica Microsystems GmbH, Wetzlar, Germany).

#### BoHV-4 replication in the genital submucosa upon direct injection

In order to investigate if fibrocytes can be infected by BoHV-4 in the genital submucosa, a direct injection was performed. Explants were injected with 0.2 mL containing 2 × 10^7^ TCID_50_ BoHV-4 mixed with 0.2 μL carboxylate-modified microspheres [FluoSpheres^®^, 0.2 μm, red fluorescent [580/605] (Invitrogen no. F8810)] or PBS alone [28]. A 1 mL syringe and a thin needle (0.9 × 25 mm) were used. The explant was further cultured at 37 °C and then collected at 72 hpi. Cryosections (20 μm) were made. The above virus staining protocol was used. Analysis was performed by TCS SPE confocal system.

### Statistical analysis

Data were statistically processed by Graphpad Prism 5.0 (GraphPad Software, Inc., San Diego, CA, USA) for analysis of variance (one-way ANOVA). The data are represented as means with standard deviation (SD) of three independent experiments. Results with *P* values of ≤ 0.05 were considered significant.

## Results

### Tissue viability

The percentages of TUNEL positive cells in the different tissues at different time points of culture are shown in Table 1. During 96 h of in vitro culture, no significant changes were found in the occurrence of apoptosis in the epithelium and lamina propria (Additional file 1). The percentage of apoptotic cells in the epithelium of all tissues ranged from 0.4 ± 0.4 (0 h) to 2.8 ± 1.4 (96 h), and in the lamina propria from 0.8 ± 0.3 (0 h) to 8.6 ± 2.3 (96 h).

**Table 1:** Occurrence of apoptosis in epithelium and lamina propria as a parameter for the effect of in vitro culture on the viability of bovine genital mucosae explants

Tissue	Layer	% of TUNEL-positive cells at …h of cultivation				
		0	24	48	72	96
Vagina	Epithelium	0.8 ± 0.8	1.0 ± 0.7	1.4 ± 1.3	1.8 ± 1.3	2.0 ± 1.6
	L. propria	1.2 ± 0.8	2.6 ± 1.8	4.0 ± 1.6	5.4 ± 2.1	7.8 ± 2.4
Cervix	Epithelium	0.6 ± 0.5	2.2 ± 1.3	2.0 ± 1.2	2.4 ± 1.1	2.8 ± 1.4
	L. propria	2.6 ± 1.1	6.2 ± 2.4	6.6 ± 2.9	7.6 ± 2.9	8.6 ± 2.3
Uterus	Epithelium	0.4 ± 0.4	0.8 ± 0.4	1.2 ± 1.2	2.3 ± 1.1	1.8 ± 1.4
	L. propria	0.8 ± 0.3	1.6 ± 0.6	2.6 ± 1.8	4.6 ± 2.8	4.8 ± 2.2

Values are given as mean ± SD.

### Determination of extracellular virus titers (virus production)

**Figure 1:**
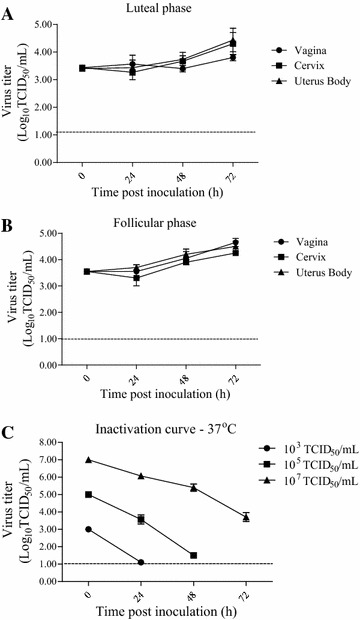
**Virus production and shedding of BoHV-4-inoculated genital explants from animals in the luteal (A) and follicular (B) phase and BoHV-4 inactivation curves (C) at 37** **°C in explant medium.** The horizontal dotted line represents the detection limit for the titration assay.

The supernatants of inoculated mucosa explants were collected and titrated at different time points in order to get insights in virus production and shedding. The virus titer curves are given in Figure 1. The virus titers increased over time from 0 h to 72 hpi in all genital tract tissues. A similar replication was observed for the cervix and uterus explants from animals in luteal and follicular phase. Concerning the vagina mucosa, although there are some differences between the luteal and follicular phase, no significant difference was observed.

### BoHV-4 replication pattern

**Figure 2:**
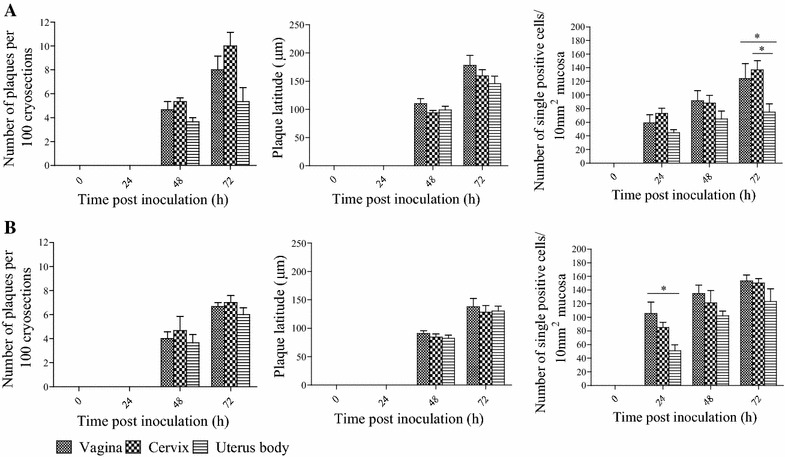
**Evolution of plaque number, plaque latitude and number of single BoHV-4 infected cells in both epithelium and lamina propria of genital tract mucosa explants from animals in the luteal (A) and follicular (B) phase**. All data represent means + SD of triplicate independent experiments and *P* values for statistical significance. Asterisks indicate statistically significant differences (≤ 0.05).

BoHV-4 positive cells were visible at all collected time points post inoculation. At 24 hpi, single positive cells or clusters of a few cells were observed in the epithelium and lamina propria. BoHV-4 positive plaques were detected in the epithelium at 48 and 72 hpi in all the genital tract mucosa explants and at both reproductive cycle phases (Figure 2).

For the luteal phase, the average number of plaques in explants from vagina, cervix and uterus was 4.7, 5.3 and 3.7/100 cryosections at 48 h and 8, 10 and 6.7/100 cryosections at 72 hpi, respectively. The BoHV-4-induced epithelial plaques significantly increased in plaque latitude over time in all genital tracts. The viral plaque latitude in the vaginal mucosa increased significantly over time from 48 (112.4 ± 26.6 μm) to 72 hpi (178.3 ± 57.2 μm), in the cervix mucosa from 48 (94.0 ± 16.5 μm) to 72 h (159.4 ± 42.1 μm) and in the uterus from 48 (99.2 ± 22.6 μm) to 72 h (145.7 ± 45.8 μm). The number of single positive cells increased significantly over time from 0 to 72 hpi in the different parts of genital mucosa. At 72 h, the number of single positive cells in vagina and cervix was 124 and 137/10 mm^2^ mucosa, respectively, which was significantly higher than that in the uterus body (75/10 mm^2^ mucosa).

For the follicular phase, the number of plaques in vagina, cervix and uterus body was 3.3, 4.7 and 3/100 cryosections at 48 h and 7.7, 7 and 5.3/100 cryosections at 72 hpi, respectively. The viral plaque latitude in the vaginal mucosa increased significantly over time from 48 (90.7 ± 16.2 μm) to 72 hpi (137.5 ± 44.0 μm), in the cervix mucosa from 48 (84.3 ± 20.5 μm) to 72 h (128.4 ± 38.0 μm) and in the uterus mucosa from 48 (82.3 ± 17.5 μm) to 72 h (130.3 ± 40.9 μm). The number of single positive cells in the vagina was 152/10 mm^2^ mucosa, which was significantly higher than that in the uterus (51/10 mm^2^ mucosa) at 24 hpi. No significant difference was detected between 48 and 72 hpi.

**Figure 3:**
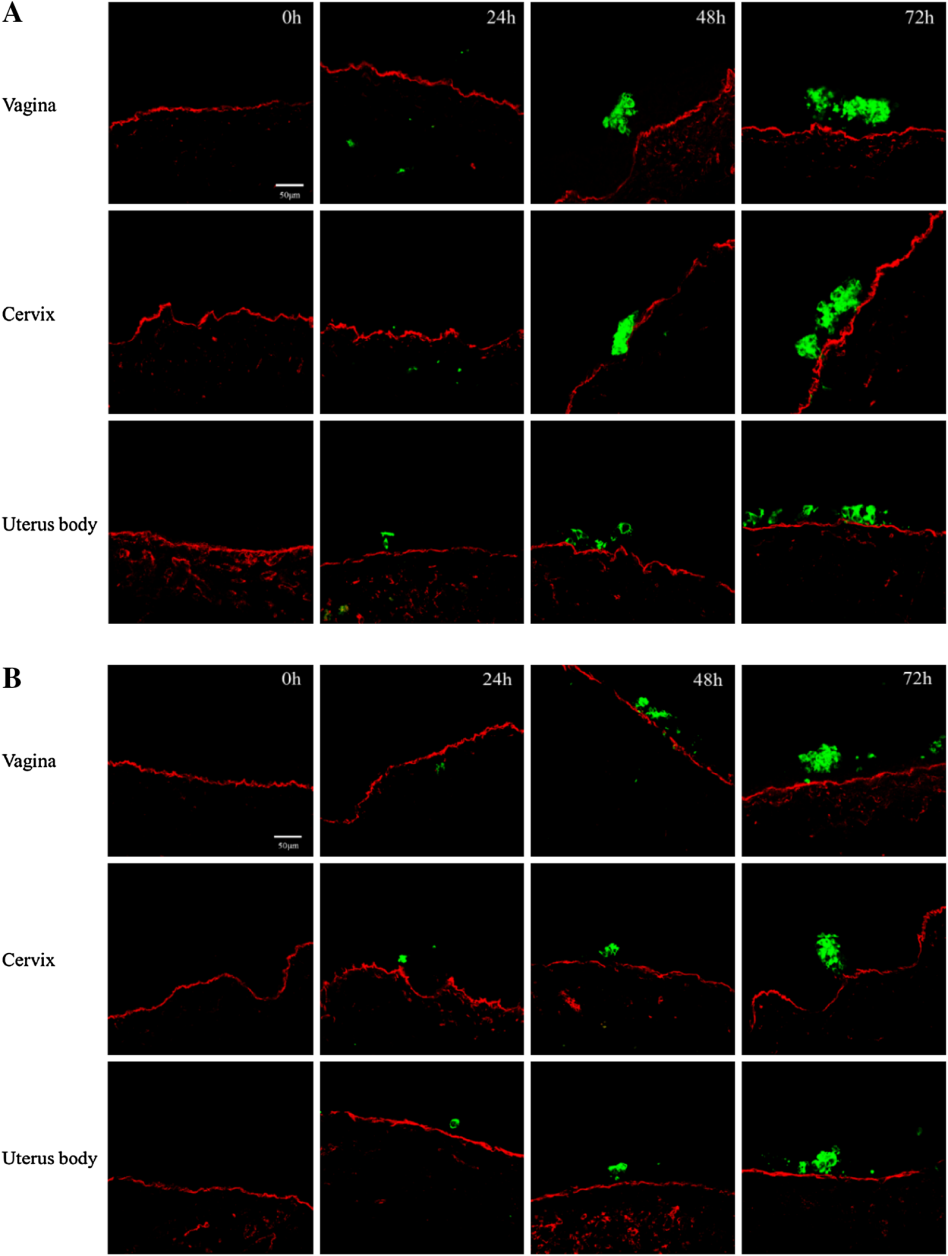
**Evolution of BoHV-4 infection in bovine genital mucosa explants at 0, 24, 48** **h and 72** **hpi.** Representative micrographs of BoHV-4 replication at 0, 24, 48 h and 72 hpi in the genital mucosa from animals in the luteal (**A**) and follicular (**B**) phase. Red represents the basement membrane and green represents BoHV-4.

BoHV-4-induced plaques did not cross the basement membrane (BM) of all tissues and both phases at any time point post inoculation (Figure 3).

The number of plaques, plaque latitude and infected cells in the genital tract were compared between animals in the follicular phase and animals in the luteal phase. The number of plaques in the luteal phase was slightly but not significantly higher than that in the follicular phase. The plaque latitude in the luteal phase was also higher but not significantly higher than that in the follicular phase.

### BoHV-4 cell tropism at 24 h after inoculation

**Figure 4:**
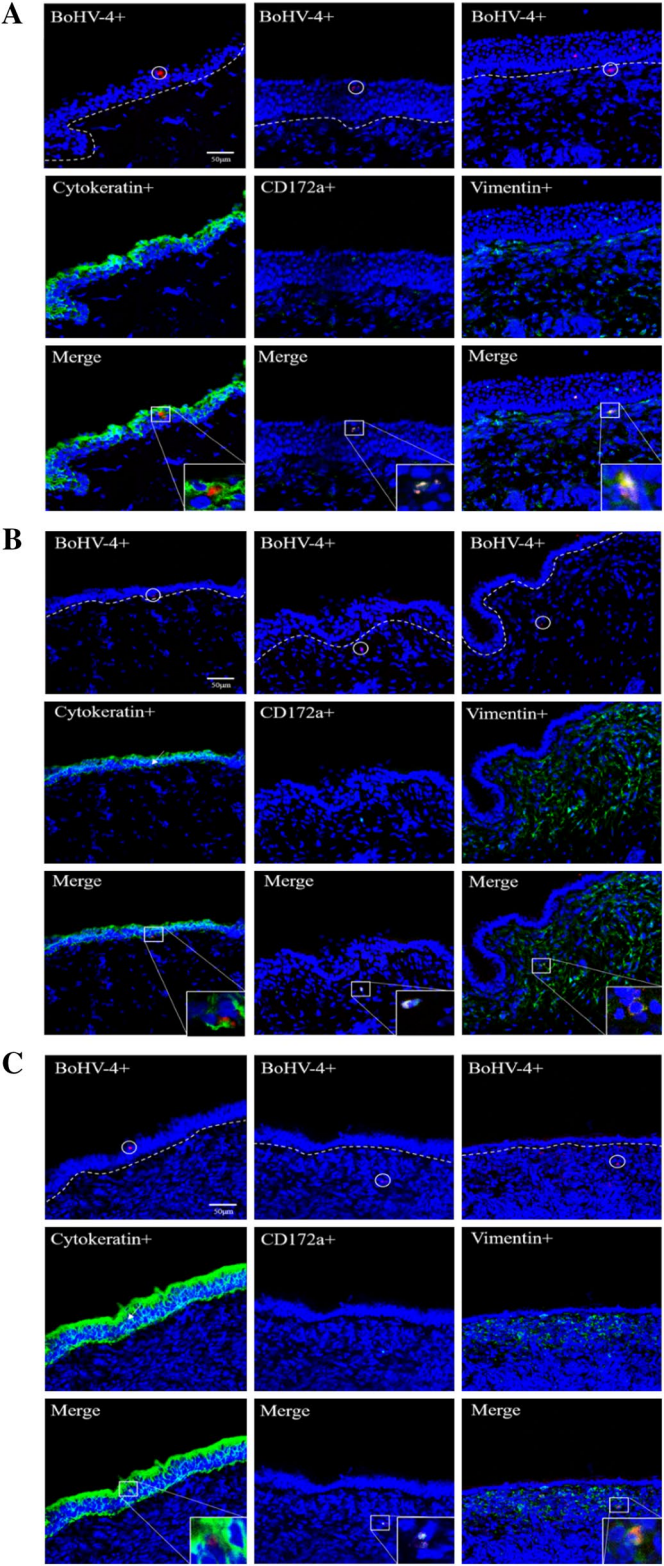
**Cell tropism of BoHV-4 at 24** **hpi in different parts of the genital mucosa [vagina (A), cervix (B) and uterus (C)] from animals in the luteal phase.** The top row shows BoHV-4 positive cells (Alexa fluor^®^ 594) within the epithelium (dotted line = basement membrane). The middle row shows respectively cytokeratin, CD172a and Vimentin expression (FITC). The bottom row represents merges of the rows above.

To better understand the cell tropism of BoHV-4 after inoculation, immunofluorescent double stainings were performed on tissues of the genital tract from the three different animals in the luteal phase at 24 h (Figure 4). The majority of infected cells in the epithelium were epithelial cells. The percentage of infected cells that were identified as cytokeratin^+^ epithelial cells in vagina, cervix and uterus body was 88.9 ± 8.6, 85.7 ± 9.8 and 79.5 ± 13.3%, respectively. A minority of infected cells were characterized as CD172a^+^ monocytic cells in the epithelium. In the lamina propria of vagina, cervix and uterus 83.2 ± 16.4, 71.3 ± 12.1 and 79.0 ± 10.5% of the infected cells were vimentin^+^ and 55.0 ± 16.2, 38.7 ± 22.8 and 46.2 ± 21.9% infected cells were found to be CD172a^+^. Neither BoHV-4 infected B nor T lymphocytes were observed at 24 hpi (Table 2).

**Table 2:** Identification of BoHV-4 infected cells at 24 h post inoculation in genital tissues

Tissue	Zone	Marker-positive BoHV-4-infected individual cells in 20 cryosections/total number of infected cell (%)				
		Cytokeratin^+^	CD172a^+^	IgM^+^	CD3^+^	Vimentin^+^
Vagina	Epithelium	88.9 ± 8.6	5.1 ± 3.8	0.0 ± 0.0	0.0 ± 0.0	8.3 ± 2.1
	L. propria	0.0 ± 0.0	55.0 ± 16.2	0.0 ± 0.0	0.0 ± 0.0	83.2 ± 16.4
Cervix	Epithelium	85.7 ± 9.8	10.3 ± 5.4	0.0 ± 0.0	0.0 ± 0.0	12.8 ± 6.1
	L. propria	0.0 ± 0.0	38.7 ± 22.8	0.0 ± 0.0	0.0 ± 0.0	71.3 ± 12.1
Uterus	Epithelium	79.5 ± 13.3	15.4 ± 12.6	0.0 ± 0.0	0.0 ± 0.0	10.6 ± 4.3
	L. propria	0.0 ± 0.0	46.2 ± 21.9	0.0 ± 0.0	0.0 ± 0.0	79.0 ± 10.5

### BoHV-4 replication in the genital submucosa upon direct injection

**Figure 5:**
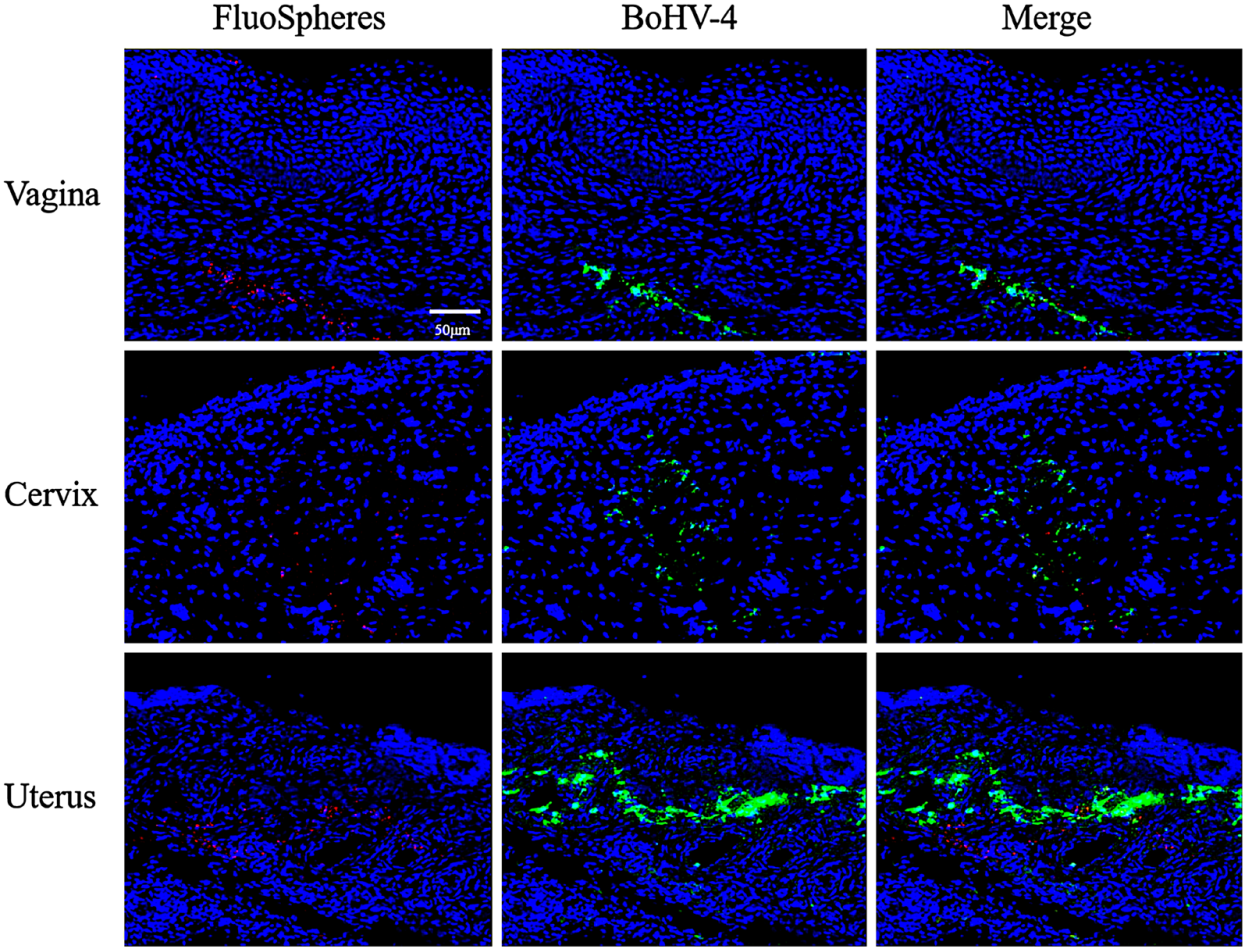
**BoHV-4 replication in bovine genital mucosa explants at 72** **h after direct injection in the lamina propria.** Red represents the FluoSpheres and green indicates the BoHV-4 infected cells.

After injection of a mixture of carboxylate-modified microspheres and BoHV-4, an immunofluorescent staining was performed on different genital mucosae from animals in the luteal phase to detect viral replication in the connective tissue at 72 hpi (Figure 5). At the injection site (red beads), plaques of BoHV-4 infected cells (green) were found in the submucosa of vagina, cervix and uterus, which indicates that the virus can easily replicate in the fibrocytes underneath the epithelium.

## Discussion

Until now, a lot of work has already been done on the pathogenesis of gammaherpesviruses. However, data concerning the primary replication and dissemination at the host mucosal entry ports are scarce. Getting insights into how the virus behaves at its primary replication site is important because it can provide methods for prevention and treatment of the viral infection in a rational way. Previously, a murine in vivo model was established which allowed to study the sexual transmission of Murid Herpesvirus 4 (MuHV-4). With this model, Francois et al. were able to demonstrate that MuHV-4 is genitally excreted after latency establishment in intranasally infected female mice. Additionally, efficient virus transmission was observed from females to males following sexual contact and was mediated by the envelope glycoprotein gp150 of MuHV-4 [29, 30]. In this study, tissues from vagina, cervix and uterus body of cows in the luteal and follicular phase were used to establish bovine genital mucosa explants in order to examine the early events of the pathogenesis of a BoHV-4 infection.

Single BoHV-4 infected cells or small clusters were already present at 24 hpi and the majority of the infected cells were of epithelial origin. Only from 48 hpi, BoHV-4 positive plaques were visible in the epithelium. BoHV-4 positive plaques did not cross the basement membrane, which is in contrast with the bovine alphaherpesvirus, bovine herpesvirus 1 (BoHV-1). Previous work in our laboratory has shown that BoHV-1 is capable of drilling its way through the basement membrane in an aggressive way. Likewise, other alphaherpesviruses, such as herpes simplex virus-1, pseudorabies virus and feline herpesvirus-1 exhibit also a plaquewise spread through the BM of genital mucosa explants [31–34]. When compared with BoHV-1, BoHV-4 replicated slower in genital explants.

Concerning the BoHV-4 replication in the genital tract of animals in the follicular phase with that of animals in the luteal phase, the number of plaques and plaque latitude in the luteal phase were slightly larger than that in the follicular phase. This might imply that during the luteal phase the genital tract is more susceptible for BoHV-4 than that during the follicular phase. This could be due to a less effective immunity. Indeed, recent studies indicate that progesterone reduces the number of immune cells in the genital tract and inhibit cytokine production by blood mononuclear cells [35–37]. Thus, the higher the progesterone concentration, the stronger the inhibition of the innate immune response. Progesterone is significantly higher during the luteal phase than during other phases of the reproductive cycle, which might explain the more extensive infection of BoHV-4 in genital tract explants as a result of the lower innate immune response. Multiple studies have shown that female sex hormones have a profound effect on the susceptibility to genital herpes infection [29, 38–40]. Francois et al. examined the MuHV-4 shedding from the vagina and revealed that this excretion was dependent on the presence of estrogens [29]. In contrast, the presence of progesterone has been shown to increase the susceptibility of the genital tract to herpes simplex virus type 2 (HSV-2) infection [41]; the presence of estrogen decreased the risk of HSV infection in the female genital tract [42–44]. The present study revealed a slight difference in viral infection of explants obtained during the luteal and follicular phase of the menstrual cycle, however, the observed difference was not significant. Because the genital explants were not treated with hormones during the in vitro cultivation, the effects may have been reduced.

The number of single infected cells in the uterus was significant lower than that in the vagina and cervix. This result indicates that BoHV-4 infection in the uterus might be less efficient than in the vagina and cervix. According to the staining results, the number of monocytic cells in vagina and cervix is significantly higher than that in the uterus (data not shown). The smaller population of monocytic cells in the uterus might explain the lower infection.

Although BoHV-4 induced epithelial plaques do not cross the basement membrane, several single positive cells were found in the lamina propria, underneath the basement membrane. To identify which cells are infected, a double staining for cell type and virus was performed. The results showed that the majority of BoHV-4 positive cells were vimentin^+^, of which half of them were CD172a^+^. The other half did not consist of T- and B-lymphocytes. To further investigate whether fibroblasts could be infected, the explants were directly injected with BoHV-4. The results showed that viral plaques were present at the injection sites of lamina propria at 72 hpi, which demonstrated that the fibrocytes are fully susceptible for BoHV-4 replication.

Interestingly, Gaspar et al. recently found and proposed that MuHV-4 firstly infects epithelial cells, then myeloid cells and only afterwards B cells by using a homologous mouse model [45, 46]. B cell infection for MuHV-4 is described to be a late event in the pathogenesis. Indeed, Frederico et al. identified a binding block to B cell infection that was overcome by co-culture with virus propagated in myeloid cells [46]. Nevertheless, in vivo studies have shown that in persistently/latently infected cattle and rabbits especially splenic cells which are located in the marginal zone and belong to the non-T and non-B cell compartment, harbor BoHV-4. This indicates that cells of the monocyte/macrophage lineage are the most plausible site of persistent/latent BoHV-4 infection [12, 14, 47–49]. In support of this, Donofrio et al. have shown that cells of the monocyte/macrophage lineage support a BoHV-4 persistent infection [50].

In conclusion, the present study demonstrates that BoHV-4 easily infects the genital tract and may be transmitted in genital secretions. Primary replication starts in epithelial cells. Virus spreads laterally which results in the formation of small viral plaques. Virus is not able to directly cross the basement membrane; instead it hijacks CD172a^+^ monocytic cells. When BoHV-4 is produced in connective tissue, fibrocytes may become infected and may eventually lead to pathological processes. To our knowledge, this is the first report describing the invasion mechanism in genital explants. At present, both BoHV-4 and MuHV-4 are accessible experimental models for the hard to study gammaherpesviruses such as human Epstein–Barr virus (EBV) and Kaposi’s sarcoma-associated herpesvirus (KSHV).

## Additional file

**Additional file 1.** **TUNEL-staining of vagina, cervix and uterus body explants at 0 and 96** **h of cultivation.** The TUNEL assay revealed almost no apoptotic cells (green fluorescence) in vagina, cervix and uterus body at 0 h and only a low number of positive apoptotic cells at 96 h of cultivation.
