# Supplementary figures and images for: Primary replication and invasion of the bovine gammaherpesvirus BoHV-4 in the genital mucosae
Source: Vet Res. 2017 Nov 28;48:83. doi: 10.1186/s13567-017-0489-3 (PMC5706299; doi:10.1186/s13567-017-0489-3)

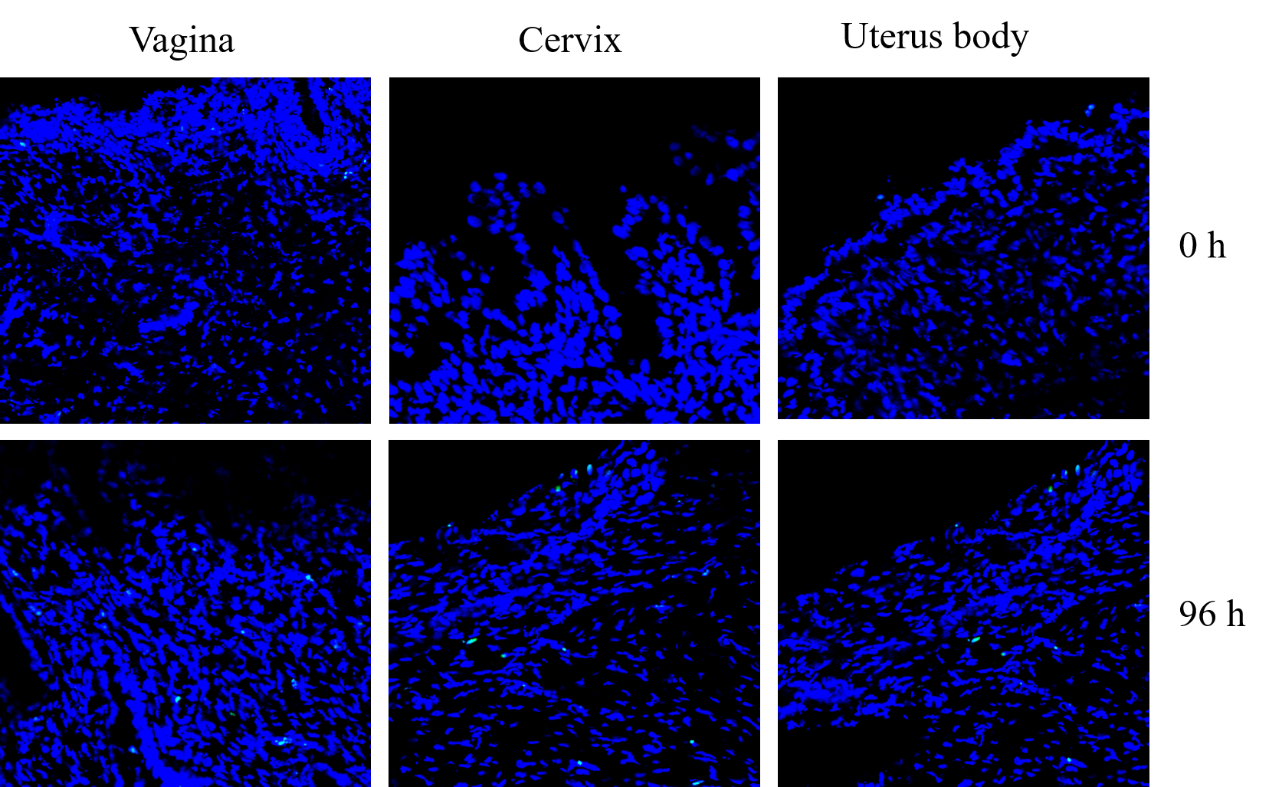

Supplement: Supplementary file 1 — Additional file 1. TUNEL-staining of vagina, cervix and uterus body explants at 0 and 96 h of cultivation. The TUNEL assay revealed almost no apoptotic cells (green fluorescence) in vagina, cervix and uterus body at 0 h and only a low number of positive apoptotic cells at 96 h of cultivation. [file 13567_2017_489_MOESM1_ESM.docx]
